# High retreatability and dimensional stability of polymer grafted waterlogged archaeological wood achieved by ARGET ATRP

**DOI:** 10.1038/s41598-019-46366-7

**Published:** 2019-07-08

**Authors:** Yihang Zhou, Kai Wang, Dongbo Hu

**Affiliations:** School of Archaeology and Museology, Peking University, Beijing, China

**Keywords:** Polymer chemistry, Polymer chemistry, Materials science, Materials science

## Abstract

To explore new methods to maintain the dimensional stability of waterlogged archaeological wood after drying and keep the natural cell lumens unaltered for future retreatments, activator regenerated by electron transfer (ARGET) atom transfer radical polymerization (ATRP) is employed to consolidate archaeological wood. To prepare the ATRP process, the waterlogged archaeological wood samples (*Pinus* massoniana with maximum moisture content of around 529%) were first modified by 2-bromoisobutyryl bromide in CH_2_Cl_2_ to acquire C-Br bonds as initiators. Then, butyl methacrylate or styrene was polymerized to the remaining cell walls with catalyst (CuBr_2_), reductant (ascorbic acid) and ligand (PMDETA) in ethanol. After the treatment, the samples were washed and naturally dried. The results characterized by microscopy showed that the polymerization only took place within the remaining cell walls, showing no sign of collapse or distortion after air drying, and all natural cell lumens could be retained for future retreatments. Also, anti-shrinkage efficiencies as high as 87.8% for the wood sample grafted with polystyrene and 98.5% for the wood sample grafted with polybutylmethacrylate were obtained from the treatment described in this paper, indicating modification of grafting polymer through ARGET ATRP can help maintain the dimensional stability of water archaeological wood effectively.

## Introduction

Scientists and conservators have put tremendous efforts into methods aimed at maintaining the dimensional stability of waterlogged archaeological wood, preventing them from shrinkage, cracking and deformation. In early days, alum (KAl(SO_4_)_2_·12H_2_O, aluminum potassium sulphate decahydrate), drying oil, sugar and natural resins were commonly used as bulking agents for waterlogged archaeological wood. Later synthetic polymers were introduced in the middle 19^th^ century, among which the most well-known is polyethylene glycol (PEG), first largely used on the famous warship *Vasa*^1^. However, even the most widely used PEG has its deficiencies, i.e., leaching, color changing, susceptibility to various aerobic and anaerobic bacteria^2,3^ and the production of formic acid as a result of degradation^4^. Also, some of conservation materials ever thought to be reversible, like Paraloid 72, acrylic emulsion and polyvinyl acetate emulsion, have been proven not to be entirely reversible and the reversible rates decline with an increase of aging^5,6^. Removal of such conservation materials is generally difficult and probably pose new threats to the cultural heritage. However, the use of reversible consolidation materials or methods is never the only option. Recent research has proven *in situ* polymerization of isoeugenol is a green and promising method for waterlogged archaeological wood with potential retreatability^7^, while many oligomers were generated in solution, reducing the efficiency. A variety of organosilicon compounds, including methyltrimethoxysilane, Octyltriethoxysilane, (3-Mercaptopropyl)trimethoxysilane, etc., also shows promising results for waterlogged archaeological wood consolidation^8–10^. However, the presence of water will reduce the consolidation effect of organosilicon compounds.

**Figure 1:**
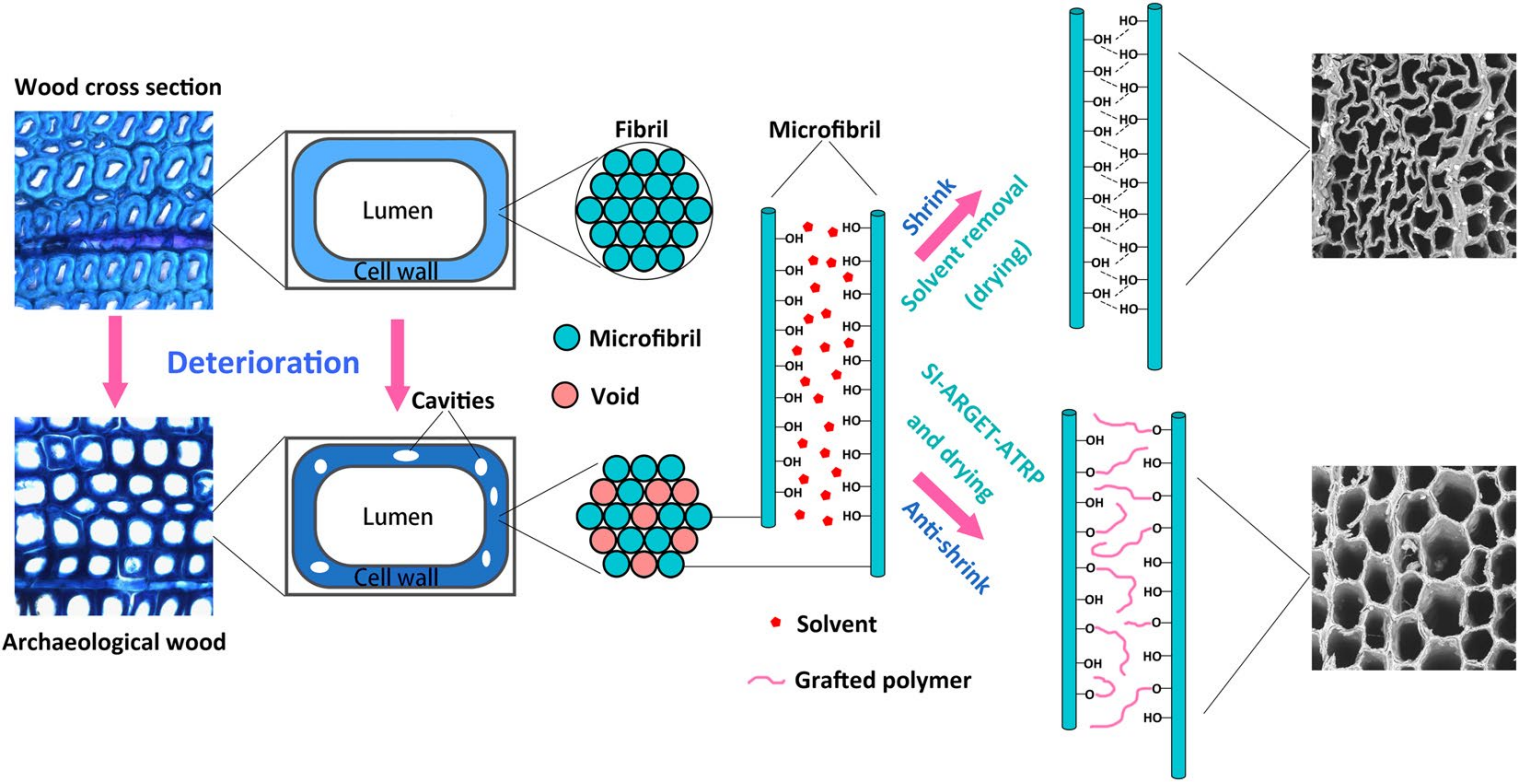
Scheme depicting the conceptual process of grafting polymer chains within wood cell wall using ARGET ATRP.

From the perspective of considering the processes responsible for the deterioration of archaeological wood, we may have a better understanding of decayed wood structures, and thus propose tailor-made methods. Microbial decay is the major cause of deterioration of wood under most circumstances. Morphological characteristics for both bacterial and fungal decays have been described in many studies^11,12^. To put it simply, either cavities within the secondary wall of fibers are formed or the secondary wall is eroded from the lumen side to the middle lamella in most cases of microbial decay. The secondary wall is known as the principle structure in fiber cells for supporting standing trees and providing strength and elasticity for the wooden object(s) later crafted from them. Therefore, the most desirable way to consolidate archaeological wood is through the restoration or reinforcement of the secondary wall. To accomplish this, *in situ* graft polymerization presents a promising approach worthy of further investigation^7,13,14^. The application of wood-polymer composites (WPC) in art conservation was proposed in the 1970s, as a method that can greatly reduce the effect of wood defects due to changes in moisture content, such as decay, shrinking and swelling^15^. However, polymerization treatments of archaeological wood are less commonly conducted because of its irreversibility. Therefore, considerable attention needs to be paid when polymerization is applied to precious archaeological wood. Theoretically, any means or material used in conservation of cultural properties should be either reversible or retreatable, in case certain materials deteriorate or might prove harmful in future. Unfortunately, conventional radical polymerization is controllable to a very limited extent and the lumens of fibers and vessels are often filled with polymer after *in situ* polymerization^13,14^. To avoid this, the polymerization process should be restricted to the remaining cell walls, which will leave very few free polymers present in cell lumens and thus the voids can be left largely unaltered to allow future retreatments. In recent research, *in situ* polymerization of isoeugenol initiated by HRP^7^ and organosilicon compounds treatments^8–10^ have achieved such a result. But we still hope to explore more methods through different polymerization strategies so that more options are available in the future research and practices. In the last few decades, reversible-deactivation radical polymerization (RDRP) or controlled radical polymerization (CRP) has developed rapidly, which provides us more controllable polymerization methods with determined polymer architectures and terminal groups. The most appropriate approach is called atom transfer radical polymerization (ATRP)^16^. This approach was revised and improved in 2006 to initiators for continuous activator regeneration (ICAR) ATRP and activator regenerated by electron transfer (ARGET) ATRP^17^. These polymerization mechanisms are described in detail in references^18,19^. Several research on grafting of methacrylate, styrene and other vinyl monomers to contemporary wood show that ARGET ATRP is a promising method for chemical functionalization of wood, such as hydrophobicity and antibacterial activity^20–22^. Moreover, ARGET ATRP allows the presence of limited amounts of air, as well as other inhibitors due to the addition of an excessive amount of reductant^23^, which greatly simplifies the reactive unit to a simple sealed container and reduces the cost of the practice. Thus, we are aiming to reinforce secondary cell walls of archaeological wood through graft polymerization using the ARGET ATRP approach (Fig. 1.) after immobilization of the initiator on wood substrate. Thereafter, it is of necessity to conduct a morphological and chemical study of the archaeological wood modified by grafting polymer through ARGET ATRP to show the integration between cell walls and polymers, and voids ready for future retreatments.

## Results and Discussion

### Chemical analysis

**Figure 2:**
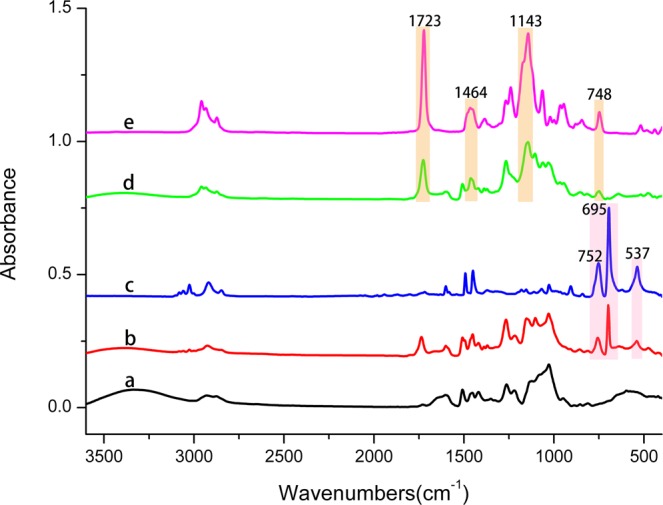
FTIR spectra of (**a**) untreated archaeological wood, (**b**) wood-g-PSt (sample PSt1), (**c**) PSt, (**d**) wood-g-PBMA (sample PBMA3) and (**e**) PBMA.

**Figure 3:**
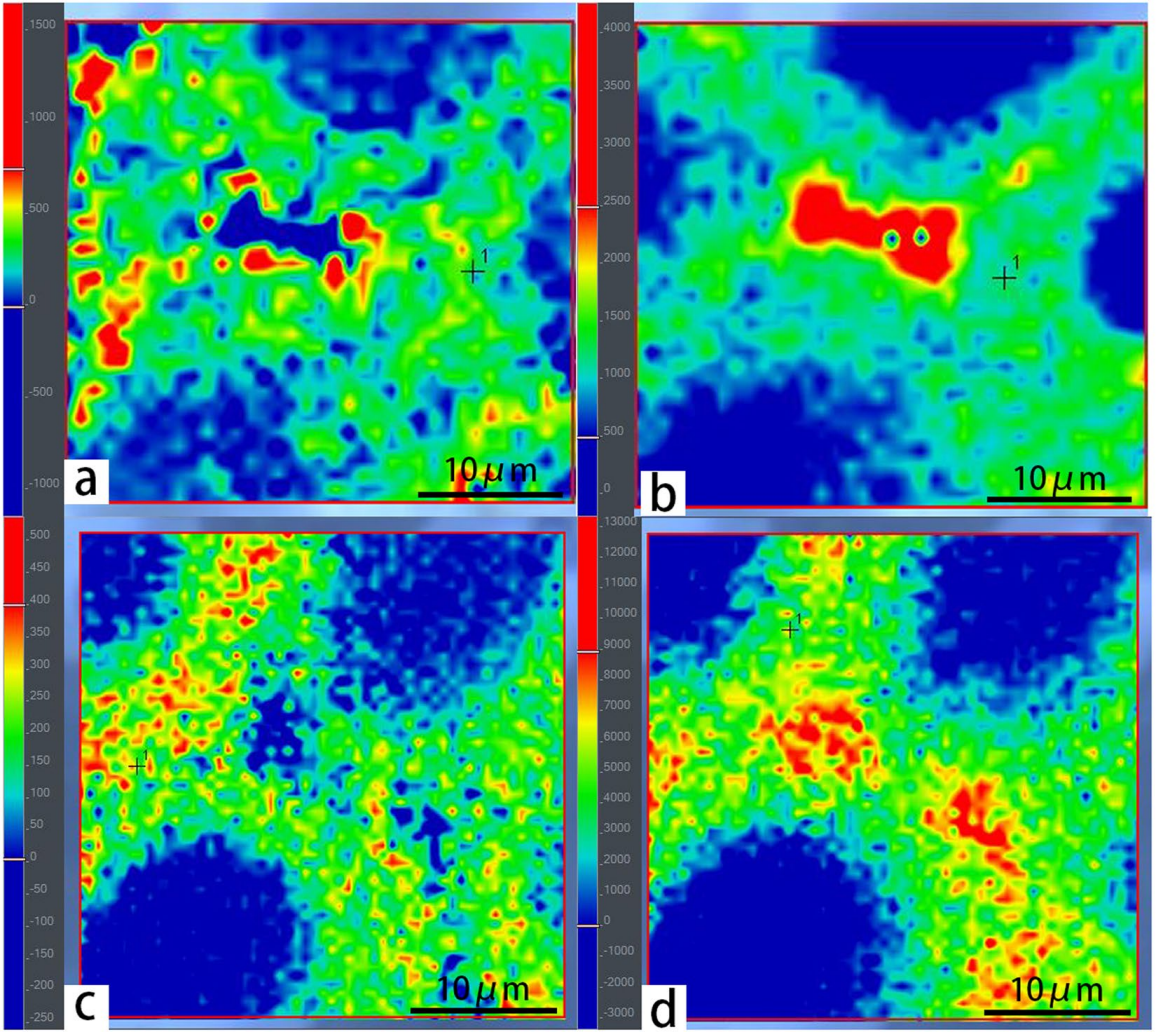
Raman imaging of (**a**) PBMA distribution of the sample PBMA4 analyzed by peak height at 847 cm^−1^, (**b**) lignin distribution of the sample PBMA4 analyzed by peak height at 1601 cm^−1^, (**c**) PSt distribution of the sample PSt2 analyzed by peak height at 1001 cm^−1^, and (**d**) lignin distribution of the sample PSt2 analyzed by peak height at 1601 cm^−1^.

FTIR analysis of untreated archaeological wood, polymer-grafted archaeological wood, poly butyl methacrylate (PBMA) and poly styrene (Pst) was conducted to confirm the presence of PBMA or PSt within the wood samples. As shown in Fig. 2, the main characteristic absorption peaks of PBMA, easily distinguishable from those of untreated archaeological wood are located at 1723 cm^−1^ (for stretching vibration of the ester carbonyl group), 1464 cm^−1^ (for bending vibration of the –CH_2_-group), 1143 cm^−1^ (for stretching vibration of the C–O–C group), 748 cm^−1^ (for bending vibration of the –CH_2_- group), etc. These peaks also appear in the spectrum of wood grafted with PBMA sample (wood-g-PBMA), which indicates that PBMA was grafted from the wood substrates through ARGET ATRP since very few free polymer chains could generate in this ATRP system. Also, PSt was successfully grafted from the wood substrates, which is determined by the absorption peaks at 752 cm^−1^ and 695 cm^−1^ (for bending vibration of the –CH_2_- and –CH- groups from mono-substituted benzene ring). Compared to untreated archaeological wood, the alteration of absorption peaks within the ranges from 3100 cm^−1^ to 2800 cm^−1^ and from 1600 cm^−1^ to 1000 cm^−1^ can also be noticed after the polymerization procedure. In addition, the absorption at 1726 cm^−1^ in Fig. 2b caused by the ester carbonyl group confirms the modification of 2-bromoisobutyryl bromide (BIBB). To further analyze the distribution of the polymer grafted from the cell wall, Raman imaging was adopted, shown in Fig. 3.

Due to deterioration, the main component of archaeological wood is lignin, of which the highest Raman shift peak is at 1601 cm^−1^. The distributions of lignin of both PBMA4 and PSt2 samples (in Fig. 3b,d) are similar to that of natural wood, i.e. more concentrated in middle lamella and corners than in secondary walls. The distributions of grafted polymers in sample PBMA4 and PSt2 (in Fig. 3a,c), measured by their highest peaks at 847 cm^−1^ and 1001 cm^−1^ respectively, indicate the graft polymerization took place in secondary walls evenly and corners were barely affected, possibly because the corners were less porous.

### Morphology under microscope

**Figure 4:**
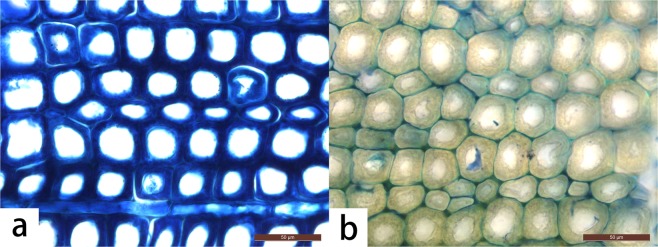
Optical micrographs of (**a**) untreated archaeological wood and (**b**) PBMA grafted archaeological wood, scale bar: 50 μm.

**Figure 5:**
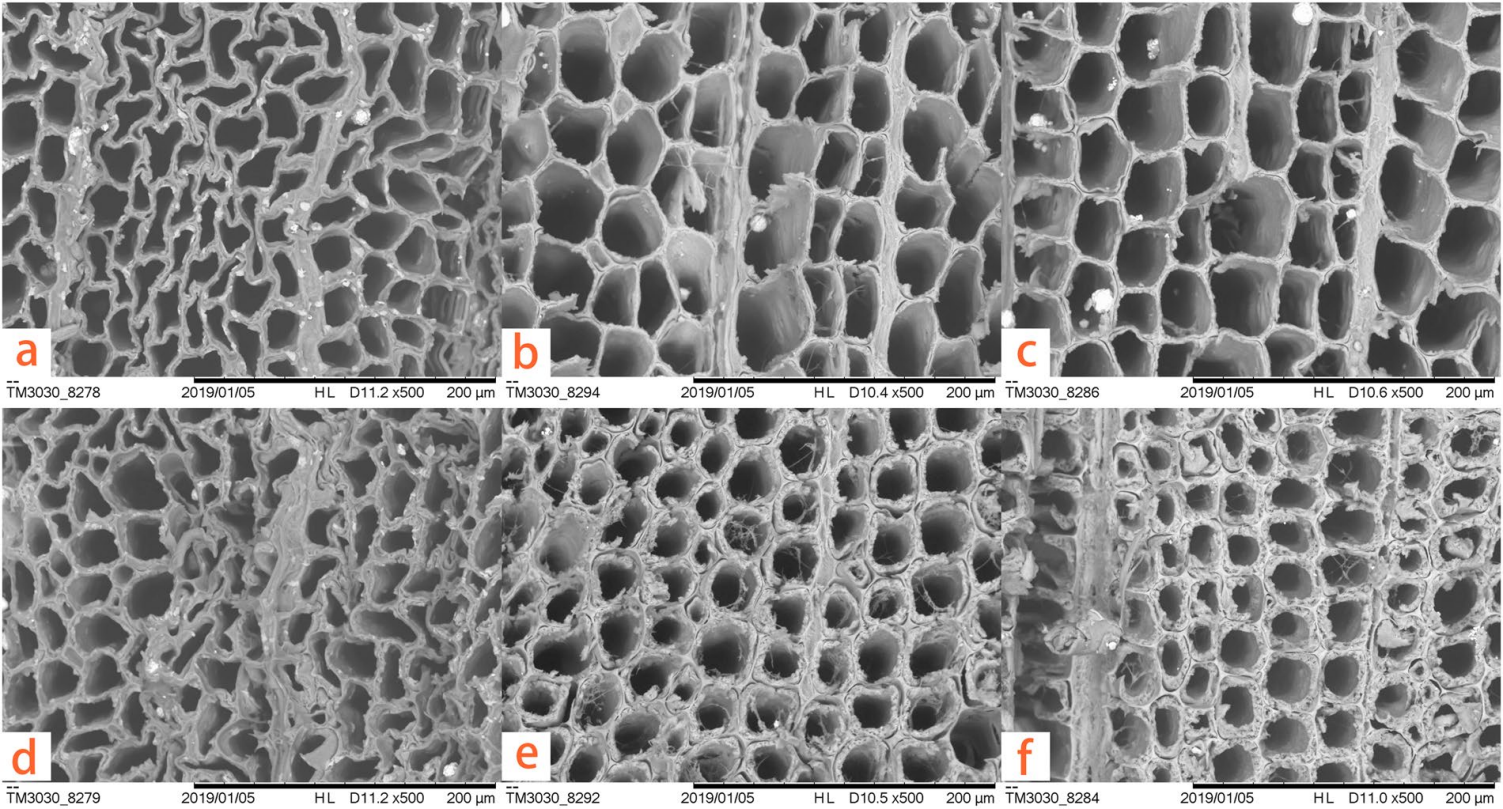
SEM micrographs of (**a,d**) untreated air-drying archaeological wood, (**b,e**) PSt grafted air-drying archaeological wood and (**c,f**) PBMA grafted air-drying archaeological wood, (**a–c**) for early wood part, (**d**–**f**) for late wood part.

**Figure 6:**
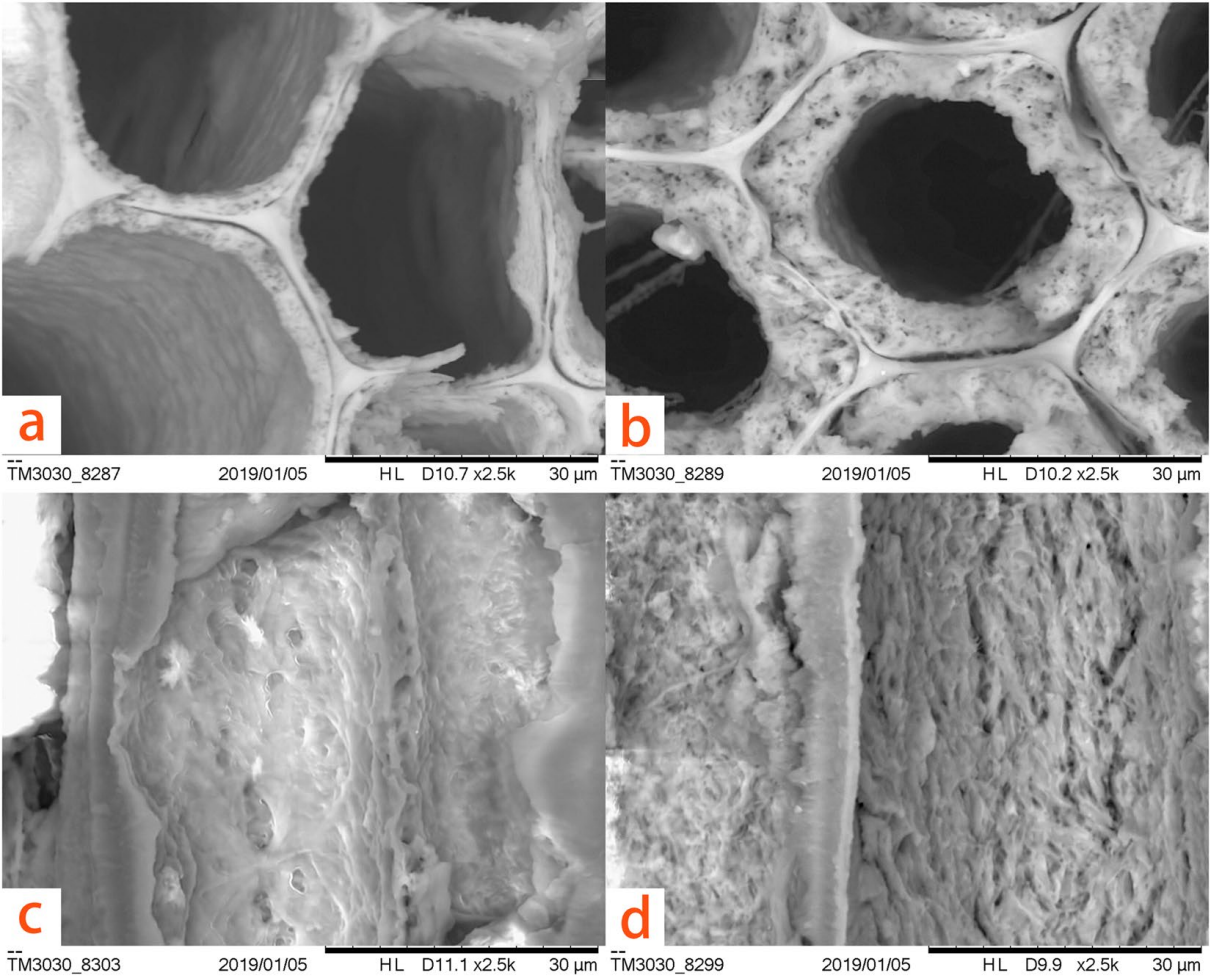
Enlarged SEM micrographs of (**a**) early wood part of the PBMA grafted sample, (**b**) late wood part of the PBMA grafted sample, (**c**) longitudinal section of the untreated sample, (**d**) longitudinal section of the PBMA grafted sample.

**Figure 7:**
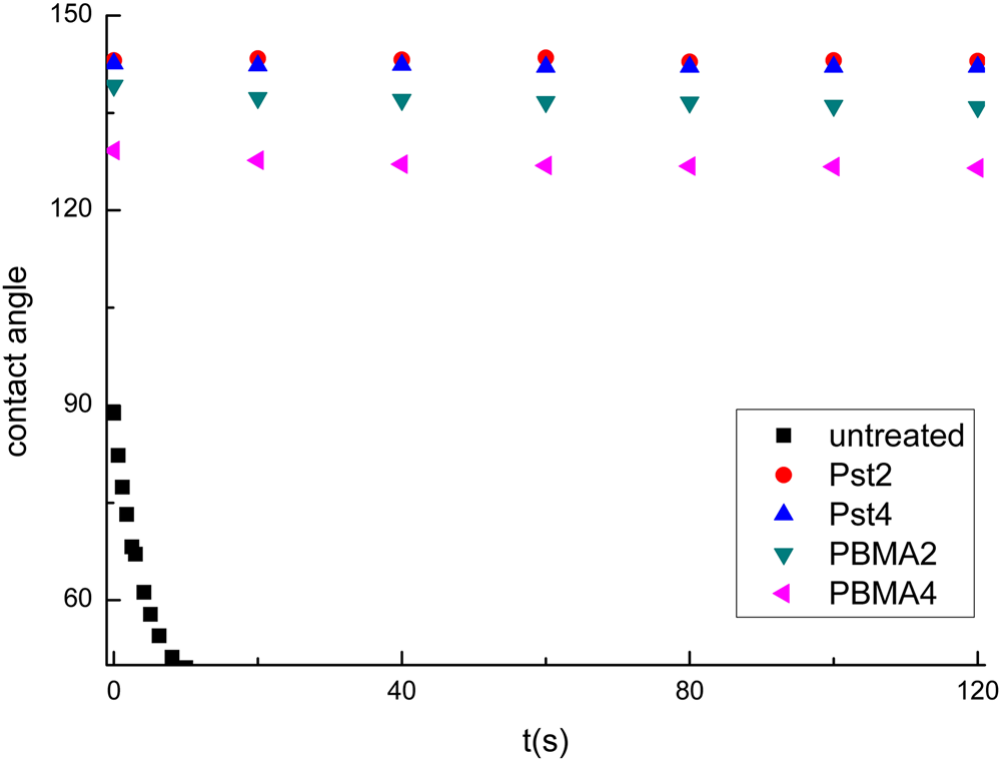
Water contact angles of the samples measured on transections.

The typical optical and SEM micrographs of both polymerized and untreated wood samples are shown in Figs 4–6. After hundreds of years of burial, the archaeological wood presents evident deterioration. The secondary wall is distinctly thinner and porous due to microbial activities and substance losses. The cavities within the secondary wall can be interpreted as the feature associated with eroding bacteria^24^. Also, when stained using toluidine blue (TB, 1% in aqueous solution), the secondary wall appears dark blue (Fig. 4a) because of residual concentrated lignin and its porous structure. After grafting PBMA by ARGET ATRP, the properties of wood specimens changed dramatically (Fig. 4b). The modified cell walls were completely chemically altered and became so highly hydrophobic that the applied TB (1% in aqueous solution) hardly wetted and stained the secondary walls, even on a freshly-cut cross section, while the ungrafted middle lamella was completely stained. This is because the cell walls of the samples have suffered bio-deterioration and lost most of their crystalline cellulose, which increased the accessibility and reactivity to the modification agent (BIBB) and monomers. The hydrophobicity after the treatment is also confirmed by water contact angle (WCA) measurements shown in Fig. 7. The water contact angles of the transections range from 126.5° to 143.1°, while untreated sample absorbed the water drop immediately. Moreover, air-dried blank samples shrunk dramatically and evidenced distortions of both the secondary wall and the middle lamella (in Fig. 5a,d). But after the graft polymerization of PBMA or PSt, no apparent shrinkage and distortion can be noticed in both early wood and late wood parts and no reductant polymer was evident in the lumens (in Figs 5b,c,e,f, 6a,b for enlarged images). Additionally, the secondary wall of air-dried untreated samples collapsed evidently resulting in the smooth inner surface while the secondary wall with grafted polymers maintained its plump state after the treatment (Fig. 6c,d). These facts strongly indicate that the ARGET ATRP process mainly took place within the secondary wall of archaeological wood as was expected and presented remarkable anti-shrinkage efficiency from the microscopic angle.

### Effectiveness of the treatment

**Figure 8:**
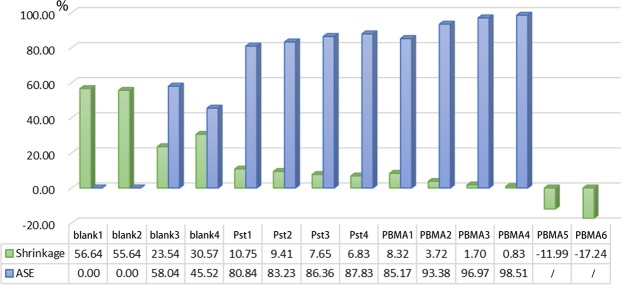
ASEs and shrinkages of the polymer grafted samples.

**Figure 9:**
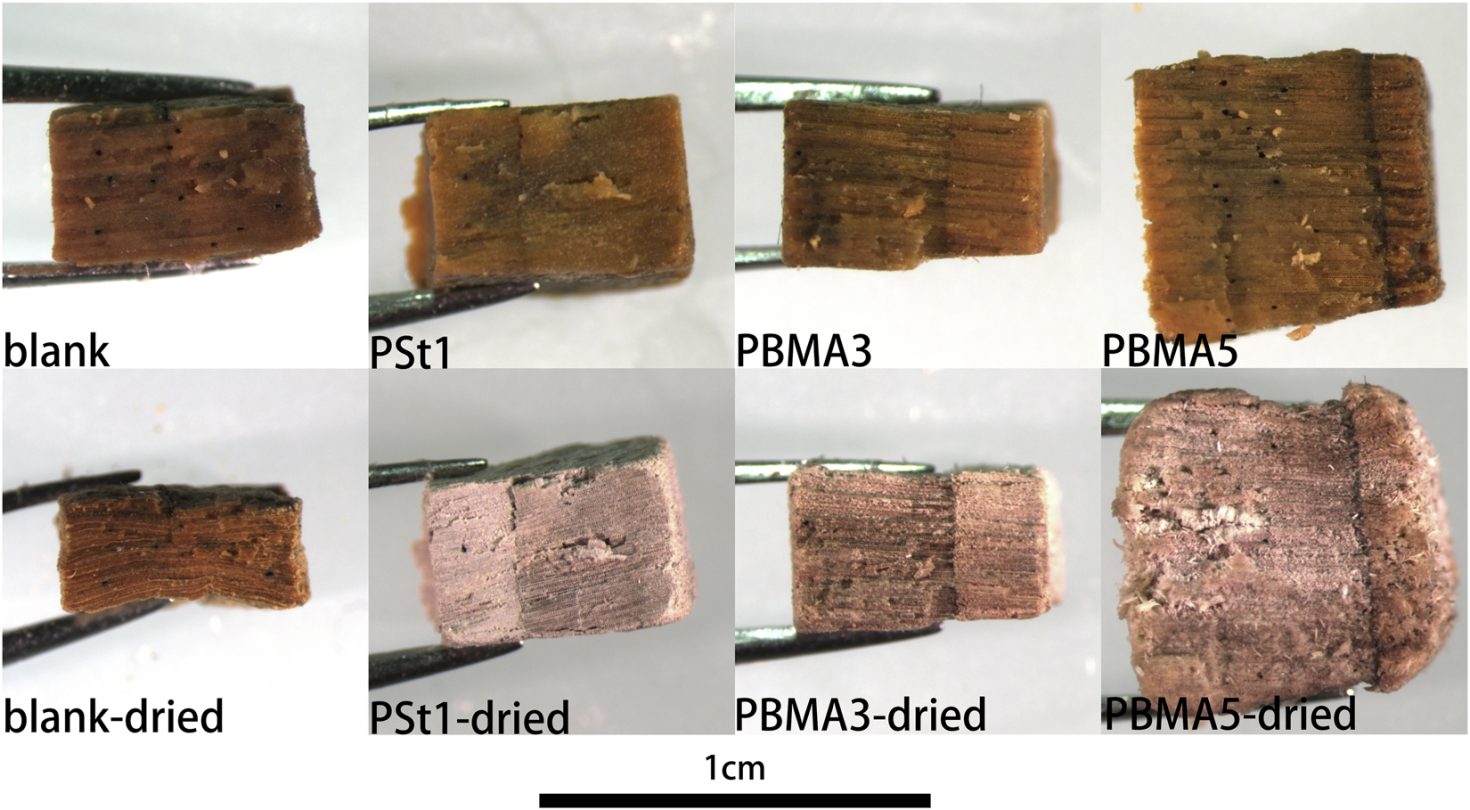
Appearance of the samples before and after the treatment and drying.

In terms of conserving waterlogged archaeological wood, the most important consideration of all is maintaining its dimensional stability, which was measured by volumetric shrinkage and anti-shrinkage efficiency (ASE) presented in Fig. 8. For the samples blank1 and blank2 dried without any treatment, the volumetric shrinkage is as high as 56%. After being modified by BIBB and logged with ethanol, the shrinkage reduced to 23.5% for blank3 and 30.6% for blank4, which was mainly contributed by ethanol of low surface tension. After 1 hours of polymerization of styrene, the volumetric shrinkage dramatically reduced to 10.8% and steadily decreased to 6.8% for 4 hours’ treatment with the 87.8% ASE. The treatment effects of wood-g-PBMA samples were even more positive with the lowest volumetric shrinkage as 0.8% and highest ASE as 98.5% for PBMA4. The appearances of the samples before and after the treatment are shown in Fig. 9. Waterlogged archaeological wood is often in dark color as blank resulted from the oxidation of lignin. After being grafted with PBMA or PSt and dried naturally, the samples became lighter in color and closer to well-preserved wood. However, the wood-g-PSt samples became whitish and thus BMA might be more suitable for waterlogged archaeological wood consolidation considering the color change and ASE. Nevertheless, despite the promising results, the time of polymerization or the concentration of monomer should be strictly controlled, or otherwise, the excessive grafted polymer would inflate the wood sample as PBMA5 presented in Figs 8 and 9.

## Conclusion

In this paper, a new potential method to consolidate archaeological wood by ARGET ATRP is described. High ASEs, up to 98.5% for the PBMA grafted sample and 87.8% for the PSt grafted sample, were obtained from modification of the remaining cell walls by grafting PBMA or PSt. And more importantly, the potential high levels of retreatability was shown in the micrographs, in that the polymerization mainly took place in the secondary walls and the natural cell lumens were retained, unaltered as intended. Also, a variety of monomers, including hydrophilic ones, can be used by this method apart from BMA and St. Furthermore, the synthesis of copolymer and modification of terminal groups to adjust the compatibility, hydrophobicity, retreatability and consolidation efficiency is possible theoretically and relatively easy to accomplish because of the ATRP mechanism. These advantages give the ARGET ATRP great potentials for future research valves on wood conservation. However, although this study shows the high potential of such an approach, there is still further research required, i.e. finding more applicable reaction systems (ideally in aqueous solution) using less harmful chemicals and designing the most appropriate copolymers for waterlogged archaeological wood before real application can be carried out.

## Materials and Methods

### Materials and reagents

Waterlogged archaeological pine wood (Pinus massoniana) were cut into blocks of 10 mm (longitudinal), ×5 mm (tangential) and ×5 mm (radial) approximately in size. The archaeological wood used was obtained from the Nanhai One shipwreck lying on the bottom of South China Sea for around 800 years with a maximum moisture content of around 529%. These sample were desalinated before the treatment. However, the indissoluble salts including calcium sulfate and ferrous sulfide can be hardly removed from the samples. Anisole(99%), copper(II) bromide(Cu(II)Br_2_, 99%) from Macklin Biochemical, 2-bromoisobutyryl bromide (BIBB, 98%) from Bide Pharmatech, N,N,N′,N″,N″-pentamethyldiethylenetriamine (PMDETA, 98%), 2-Bromo-2-methylpropanoic acid (97%) from D&B Biotech, pyridine (99%), Ascorbic acid (Vc, 99%) from Xilong Scientific, ethanol (AR) and dichloromethane (CH2Cl2) from Tongguang Fine Chemicals were used directly without purification. Butyl methacrylate (97% with 10 ppm MEHQ) and styrene (99.5%) were purchased from Aladdin and purified through an alkaline aluminum oxide column to eliminate inhibitors before use.

### Immobilization of BIBB on cell walls

Waterlogged archaeological wood was immersed in ethanol for two days and in dichloromethane for another two days to replace the water. The specimens were placed in 10 ml sealed flasks with a mixture of BIBB, pyridine and 3 ml dichloromethane and heated at 50 ml with magnetic stirring for an hour. The volume ratio of the wood sample, BIBB and pyridine was 4:3:2. Wood specimens were washed thoroughly with dichloromethane and then ethanol for 10 min for each solvent. Then the specimens were heated in ethanol at 50 °C for 12 h to completely remove any unreacted and unbounded reagents (specifically unbounded initiators). The specimens were kept in ethanol before the polymerization procedure.

### Grafting PBMA/PSt from the initiator-functionalized wood substrate

The wood specimens modified with BIBB were each placed in separate 10 ml sealed flasks and soaked in a mixture of ethanol and BMA (or St) for 12 h at room temperature to allow the monomers to disperse in wood homogenously. Thereafter, PMDETA (0.5% v/v, excessive for preventing impurities in archaeological wood from deactivating PMDETA or Cu ions), Cu(II)Br_2_ and an excessive amount of Vc were added into each sealed flask with limited amount of air (as less as possible). Six parallel experiments of grafting PBMA and four parallel experiments of grafting PSt were conducted with different reaction conditions for once as listed in Table 1 and then washed in ethanol thoroughly and finally dried at room temperature under ambient pressure until the weight reached a constant (< 0.001 g) (at least 2 days). Also, poly-butylmethacrylate (PBMA) and polystyrene (Pst) were prepared by polymerization in the same initiation conditions as above with 2-Bromo-2-methylpropanoic acid as the initiator. Precautions should be taken since the chemical used in the above two procedures are hazardous and flammable. Ventilating condition is required when chemicals are mixed and no flame shall present.

**Table 1:** Treatment conditions of the sampels.

No.	Cu (ppm)	Wood/monomer/solvent (v/v/v)	Reaction time (h)
PBMA1	30	1:0.5:10	4
PBMA2	30	1:1:10	4
PBMA3	30	1:2:10	1
PBMA4	30	1:2:10	2
PBMA5	30	1:2:10	3
PBMA6	30	1:2:10	4
PSt1	100	1:2:10	1
PSt2	100	1:2:10	2
PSt3	100	1:2:10	3
PSt4	100	1:2:10	4

### Blank reference samples

Two blank waterlogged archaeological wood samples labelled as blank1 and blank2 were dried in ambient pressure and temperature (20 °C) without any treatments. Additional two blank samples modified by BIBB and logged with ethanol, labelled as blank3 and blank4, were dried in ambient pressure and temperature without the polymerization procedure.

### Chemical characterization

Chemical composition of grinded specimens was determined by Fourier transform infrared (FTIR) spectroscopy (Thermo Fisher Nicolet IS50) with a range from 4000 cm^−1^ to 400 cm^−1^. Raman imaging was performed on Thermo Fisher DXRxi Micro Raman imaging spectrometer with 532 nm laser lamp. Prior to the Raman imaging, the samples are saturated by polyethylene glycol (PEG4000), sliced to a thickness of 12 μm, immersed in deuteroxide and covered with mica sheet.

### Morphology characterization

Stereomicroscope (Leica M80), Optical microscopy x(OMLeica DM4500P) and scanning electron microscopy (SEM, Hitachi TM3030) were adopted to fully illustrate the morphology of restored cell walls. For OM analysis, sections ready for observation were prepared by freehand and sealed in neutral resins. Because of the fragility of untreated archaeological wood, it has to be temporarily consolidated by PEG2000 to obtain an integral section (thus the section under OM didn’t appear to shrink). Toluidine blue (TB, 1% aqueous solution) was applied when necessary to determine the accessible lignin-rich area. For SEM analysis, only a plane of cross section was needed on each specimen.

### Physical measurement

The effectiveness of treatment was measured by calculating volumetric shrinkage and anti-shrinkage efficiency (AES) according to the following three equations:

- volumetric shrinkage:
  $${S}_{v}=\frac{{V}_{0}-{V}_{1}}{{V}_{0}}$$
  where *V*_0_– initial volume of a specimen i.e. the volume in waterlogged conditions, V_1_ – final volume of a specimen, i.e. the volume after air-drying for a specimen,
- volumetric AES:

$${\rm{AES}}=\frac{{S}_{u}-{S}_{t}}{{S}_{u}}$$

where *S*_*u*_ – shrinkage of untreated wood, S_t_ –shrinkage of treated wood.

The volumes of the samples were all determined by water displacement method.

### Water contact angle measurement

The fresh transections of the samples below the original surface by 1 mm were prepared for the tests. The sessile drop method was used to determine the contact angle of water by placing distilled water droplets of 5.0 μm onto the surfaces and images were taken at a certain frequency. WCA was measured by optical contact angle analyzer (DSA 30) with analyzing software produced by KRUSS.

## Data Availability

The datasets generated during the current study are available from the corresponding author on reasonable request.
